# Four-Strand Core Suture Improves Flexor Tendon Repair Compared to Two-Strand Technique in a Rabbit Model

**DOI:** 10.1155/2016/4063137

**Published:** 2016-06-30

**Authors:** Alice Wichelhaus, Sascha Tobias Beyersdoerfer, Brigitte Vollmar, Thomas Mittlmeier, Philip Gierer

**Affiliations:** ^1^Abteilung für Unfall-, Hand- und Wiederherstellungschirurgie, Chirurgische Universitätsklinik Rostock, Schillingallee 35, 18055 Rostock, Germany; ^2^Rudolf-Zenker-Institut für Experimentelle Chirurgie und Zentrale Versuchstierhaltung, Universitätsmedizin Rostock, Schillingallee 69a, 18057 Rostock, Germany

## Abstract

*Introduction*. This study was designed to investigate the influence of the amount of suture material on the formation of peritendinous adhesions of intrasynovial flexor tendon repairs.* Materials and Methods*. In 14 rabbits, the flexor tendons of the third and the fourth digit of the right hind leg were cut and repaired using a 2- or 4-strand core suture technique. The repaired tendons were harvested after three and eight weeks. The range of motion of the affected toes was measured and the tendons were processed histologically. The distance between the transected tendon ends, the changes in the peritendinous space, and cellular and extracellular inflammatory reaction were quantified by different staining.* Results*. A 4-strand core suture resulted in significantly less gap formation. The 2-strand core suture showed a tendency to less adhesion formation. Doubling of the intratendinous suture material was accompanied by an initial increase in leukocyte infiltration and showed a greater amount of formation of myofibroblasts. From the third to the eighth week after flexor tendon repair, both the cellular and the extracellular inflammation decreased significantly.* Conclusion*. A 4-strand core suture repair leads to a significantly better tendon healing process with less diastasis between the sutured tendon ends despite initially pronounced inflammatory response.

## 1. Introduction

The aim of development in flexor tendon surgery is to achieve a strong suture tendon construct that allows for early active motion. Early mobilization is known to improve the outcome of flexor tendon repairs by reducing peritendinous adhesion formation and stimulating intrinsic tendon healing [1, 2]. By active motion pull-out, forces are applied to the tendon suture so that the risk of gap formation or even rerupture increases. Quite a number of studies can be found that deal with biomechanical testing of different suture techniques and suture materials, but only a few studies deal with the in vivo behavior of these suture techniques [2–5]. A multistrand core suture with multiple locking leads to a higher strength of the suture tendon construct but at the same time it increases the volume of the repaired tendon. This could possibly lead to tendon healing disorders through increased inflammatory reaction in and around the tendon [6–10].

We therefore sought to investigate the effects of the number of suture strands on tendon healing in the early and late stage in an animal model. Pivotal question was whether differences could be discovered in vivo between two- and four-strand core suture technique regarding gap formation, adhesion formation of the peritendinous space, and inflammatory response on both the cellular and extracellular pathway.

## 2. Materials and Methods

The study was conducted on 14 adult female New Zealand white rabbits weighing 2600 to 4300 g. A test model for flexor tendon surgery is established for rabbits [11, 12]. The animals were kept under standard animal laboratory conditions meeting the guidelines of both local and national authorities providing laboratory food for rodents and water ad libitum. The project was approved by the local animal care ethics committee (Landesamt für Landwirtschaft, Lebensmittelsicherheit und Fischerei Mecklenburg-Vorpommern, file reference 7221.3-1.1-109/12).

### 2.1. Surgical Technique

Rabbits were anesthetized by intramuscular administration of ketamine hydrochloride (35 mg/kg, Pfizer, Berlin, Germany) and xylazine (5 mg/kg, Bayer, Leverkusen, Germany) under maintenance of spontaneous breathing. After sufficient depth of anesthesia was reached, an intravenous line was inserted into an auricular vein so that ketamine hydrochloride (0.3 mg/kg) and xylazine (5 mg/kg) could be reapplied if necessary. Operation time took about 40 minutes. A single-shot preoperative dose of trimethoprim (5 mg/kg, ratiopharm GmbH, Ulm, Germany) was administered for infection prophylaxis. Throughout the procedure, the animals were kept at a steady temperature of 37°C with a warming pad. The surgical procedure was performed under sterile conditions using microsurgical instruments and a 2.5-fold optical magnification.

**Figure 1:**
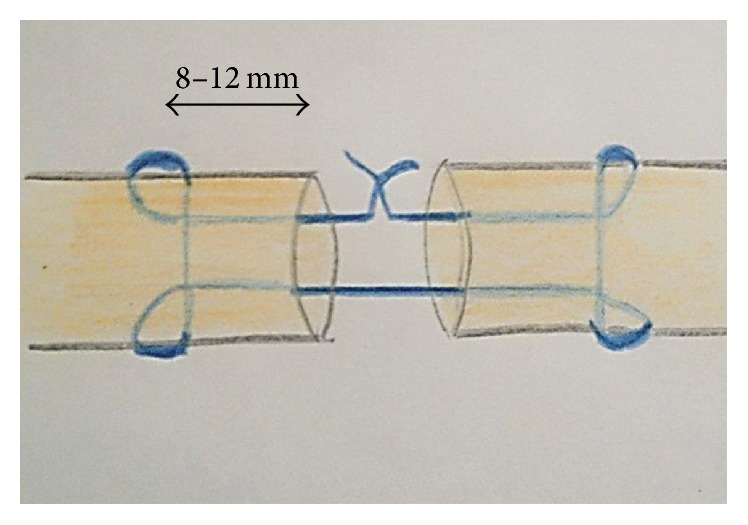
Schematic image of 2-strand core suture repair.

In a prone position the right hind leg was shaved. The surgical field was prepared with octenidine (Schülke & Mayr GmbH, Norderstedt, Germany). The skin was infiltrated with 2 mL xylocaine 1% (ratiopharm GmbH) prior to a 4 cm linear incision. The tendon sheaths of the deep flexor tendon of the third and fourth flexor tendon were incised exposing the deep flexor tendons. Both tendons were transected in a transverse way in zone II in respect to the cruciate and ring ligaments. The flexor tendon of the third digit was repaired using a modified 2-strand Kirchmayr-Kessler technique and 4-0 FiberWire (Arthrex, Naples, Florida). A scheme of the suture technique is shown in Figure 1.

On the fourth digit a 4-strand core suture was performed in analogy. A running epitendinous suture of 6-0 PDS (Ethicon, Edinburgh, United Kingdom) completed the repair. The tendon sheath was closed with a few single stitches using 6-0 PDS and the skin incisions were closed with Vicryl 4-0 (Ethicon). After skin closure, a film-forming colloidal aluminum spray was applied as wound dressing. Carprofen (5 mg/kg, Zoetis, Berlin, Germany) was applied subcutaneously as analgesic. For the first three days drinking water was supplemented with novaminsulfone (ratiopharm GmbH). Mobilization was not limited during the whole postoperative period.

### 2.2. Evaluation

To enable statements on the tendon healing process the animals were anesthetized again 3 weeks and 8 weeks, respectively, after the initial surgery. The passive range of motion of all digits was measured in comparison to the uninjured feet. Afterwards, a lethal dose of phenobarbital (ratiopharm GmbH) was applied. The flexor tendons were then harvested without disturbing the tissue architecture. In eight animals the tendons of the third and fourth digits of the left hind foot were collected as control group. Specimens were fixed in 4% buffered formalin, dehydrated and embedded in paraffin (Merck 56–58°C), and sectioned into 4 *μ*m slices longitudinally. The complete cross section including tendon and surrounding tissue was taken. Sections were stained with hematoxylin-eosin (HE) and evaluated under light microscopy (Olympus BX51, Hamburg, Germany). The tendon repair quality was quantified measuring the distance between the sutured tendon ends in 10x magnification. Gapping was classified in three groups shown in Table 1.

To evaluate peritendinous adhesion formation, the sections were examined in 400-fold magnification. The adhesion formation was classified according to Tang et al. [13]. The five different gradings are shown in Table 2.

In addition, a chloroacetate esterase (CAE) staining was realized. CAE is used to identify granulocytes from the promyelocyte stage. Hence, the complete myeloic lineage can be registered. The amount of CAE-positive cells is proportioned to the cellular inflammatory response. In 50 visual fields of each section the neutrophils were counted. Furthermore the extracellular inflammatory response was visualized via *α*-smooth muscle actin (*α*-SMA) staining. *α*-SMA is produced by myofibroblasts and released in the peri- and extracellular matrix when certain cytokines initiate the formation of myofibroblasts as a part of the response to inflammation. Therefore *α*-SMA is a marker for extracellular inflammatory response. The immunohistochemical treated sections were examined with a monitoring grid ocular at 400-fold magnification. The myofibroblast formations were counted in 5 visual fields per section and the arithmetic mean was calculated.

Histologic specimens were evaluated by an independent and study-blind pathologist.

### 2.3. Statistics

Statistical analysis was calculated with SPSS 20 software (IBM, Ehningen, Germany). A two-way analysis of variance (2-way ANOVA) was performed. Results are given as mean value ± standard deviation. *p* values less than 0.05 were considered statistically significant.

## 3. Results

All test animals survived throughout the experiment. Soon after the operation, the rabbits moved within their cages without visual impairment. Intra- or postoperative complications such as wound healing disorders and tendon rerupture were not observed. A surgical revision was not necessary in any case.

By the time of tendon harvesting all operated toes showed unrestricted passive range of motion. In the animals that were euthanized 3 weeks after the primary surgery, a significant difference was observed for tendon gapping. The tendons with 2-strand core sutures showed a mean gapping of 1.38 ± 0.27, and the tendons with 4-strand core sutures showed a mean of 0.31 ± 0.12 with a level of significance *p* = 0.02. After 8 weeks, the difference between the gapping of 2-strand and the gapping of 4-strand core sutures was still remarkable with a mean value of 1.14 ± 0.13 for the 2-strand and a mean value of 0.71 ± 0.12 for the 4-strand core sutures, *p* = 0.03.

**Figure 2:**
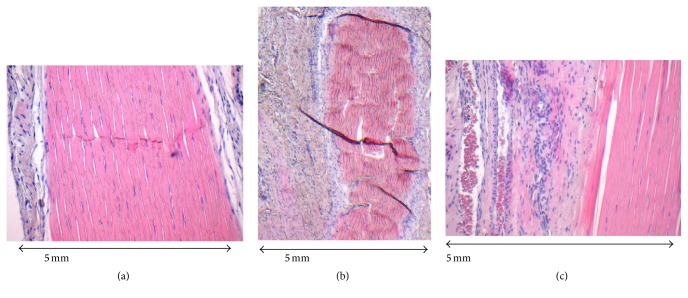
(a) HE-staining of a 2-strand core suture repair 8 weeks after initial surgery, slight peritendinous adhesion formation (20-fold magnification). (b) HE-staining of transection area of 4-strand core suture repair 3 weeks after surgery (4-fold magnification). (c) Magnification of (b) (20-fold magnification).

In all of the tendon repairs adhesive changes in the peritendinous space were discovered in the HE-stain sections.  Figure 2(a) shows HE staining of a 2-strand repair in 20-fold magnification 8 weeks after the initial surgery showing only little peritendinous adhesion formation, classified as Tang et al. stage 2.

Figure 2(b) is an image in 4-fold magnification of the transection area of a 4-strand core suture repair 3 weeks after primary surgery. The irregularity of the collagen bundles is clearly evident as well as the amount of adhesion formation around the tendon repair site.

Figure 2(c) shows the same section of the tendon repair site in 20-fold magnification. The peritendinous space is completely obliterated. Adhesion formation was classified as Tang et al. stage 5.

After assigning the specimens to one of the 5 subgroups, the 2-strand core suture repairs showed a mean Tang et al. scale of 3.54 ± 1.05 at the three-week assessment whereas the 4-strand core suture repairs showed a mean Tang et al. scale of 4.15 ± 0.80. After 8 weeks, the mean values for both repairs assimilate appreciably with a mean value of 3.43 ± 1.28 for the 2-strand core suture group and 3.07 ± 1.20 for the 4-strand core suture group. In total, the 2-strand core suture repairs were classified with lower Tang et al. scales (mean value 3.48 ± 1.15) compared to the 4-strand repairs (mean value 3.59 ± 1.15). However, none of the calculated differences proved to be significant.

At the three-week assessment, more CAE-positive cells were counted in the 4-strand core suture repairs than in the repairs using half the suture strands. However, the differences between the numbers of CAE-positive cells in 4- and 2-strand core suture repairs were not significant. After 8 weeks, there was no perceivable distinction in CAE-positive cell counts. Yet a significant decrease in the numbers of inflammatory cells occurred between the early healing phase after 3 weeks and the later phase after 8 weeks with a *p* value = 0.001 (Table 3).

Similar findings were observed by the *α*-SMA immunohistochemical staining, used to quantify the extracellular inflammatory response. In the 4-strand core suture repairs, higher *α*-SMA expressing cell counts were stated than in the 2-strand repairs. At the assessment after 8 weeks, there was no difference between the *α*-SMA expressions of both repair groups. The differences between 2- and 4-strand core sutures were not statistically significant, whereas the decrease in *α*-SMA expression from three to eight weeks proved to be significant with *p* value = 0.02 (Table 4).

**Figure 3:**
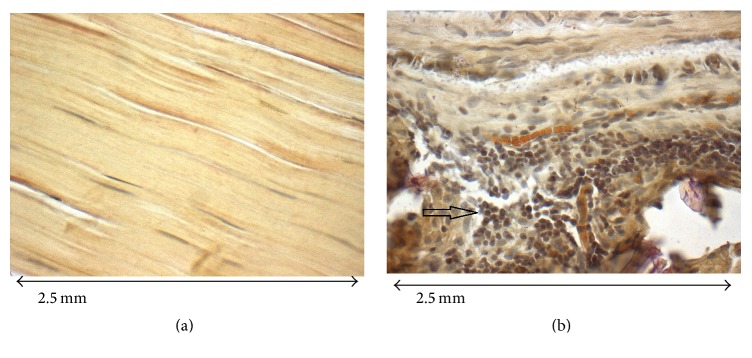
(a) *α*-SMA immunohistochemical staining of native tendon (40-fold magnification). (b) *α*-SMA immunohistochemical staining of a 4-strand core suture tendon repair site with clusters of myofibroblast formation, arrow indicating clusters of myofibroblasts (40-fold magnification).

Figure 3(a) shows a light-microscopic image of a native tendon with *α*-SMA stain in 40-fold magnification and illustrates the regular collagen fibres.

Figure 3(b) shows the *α*-SMA immunohistochemical staining of a 4-strand core suture tendon repair site after 3 weeks with clusters of myofibroblast formation in 40-fold magnification. The arrow indicates clusters of myofibroblasts. The collagen bundles are irregular with loosened structure.

## 4. Discussion

Passive-dynamic treatment has been the standard aftercare protocol for a long time. In recent years, an active early mobilization of the injured tendon is publicized increasingly as new suturing techniques showed a higher primary stability of the tendon-suture-construct in biomechanical models [2, 3, 7–10]. All of these techniques involve the insertion of more suture material into the tendon. The aim of this study was to investigate whether the amount of suture material has an impact on tendon healing in vivo.

Several suture materials and techniques have been examined regarding their tensile force. It could be shown that the biomechanical stability increases with the number of core sutures [2, 3, 8, 10]. The strength of the suture construct can be augmented by interlocks in cross-stitch or loop shape [2, 6, 14]. With regard to the suture material FiberWire proved to have pronounced rigidity over Ethilon or Prolene (Ethicon, Edinburgh, United Kingdom) [14–17]. In a preliminary study, Prolene (Ethicon, Edinburgh, United Kingdom) was used as material for the core sutures, but the threads had pulled out of the tendon as early as one week after tendon repair. Therefore FiberWire 4.0 was used in the main study, although this suture material exhibits greater resistance while passing through the tendon than monofilament suture material does despite its polished coating. The formation of foreign body granulomatous reaction has been reported using FiberWire for tendon repair [18].

The ideal suture should have sufficient tensile strength to allow an active early functional treatment, prevent intrasynovial adhesions, and promote the recovery of the sliding layer. A modified Kirchmayr-Kessler suture technique was chosen because it is technically easy and reproducible, requires little manipulation of the tendon, and has been demonstrated to have sufficient tear resistance in biomechanical studies [4, 7]. The distance of the anchor of the core suture from the cut tendon end has a major impact on the pull-out resilience of the suture. The optimum distance varies between 0.7 and 1 cm [19]. In the present study the anchor for the core suture was placed 8–10 mm from the transection site and a “locking” technique instead of a “grasping” technique was selected to reduce the danger of suture pull-out. In addition a running epitendinous circular suture was used to improve the slippage of the tendon. This circular suture not only is responsible for part of the suture's strength but also allows for securely placing the core suture's knot under the tendon surface [20].

At the three-week assessment slightly more peritendinous adhesions were found in the 4-strand core suture group than in the 2-strand core suture group in HE staining. This small difference was hardly detectable after 8 weeks, so that the number of inserted core suture strands does not seem to have a relevant influence on adhesion formation. Interestingly, the dimension of adhesion formation did not decrease significantly after 8 weeks compared to the values after 3 weeks. A progression of peritendinous adhesion could not be observed. But despite unrestricted movement of the tendons under full weight the visible changes in the peritendinous space of the repaired tendons did not vanish as expected 8 weeks after trauma induction.

Investigations have shown that leukocyte infiltration reaches a peak in the first three days after an operation and decreases to about one-third of the initial value in the first three weeks [5]. After another 5 weeks, this value halved again [5]. In this study a greater amount of CAE-positive cells was found 3 weeks after trauma, cell counts dropping significantly until the eighth week. Doubling of the core suture material showed a discreet, albeit not statistically significant, increase in CAE-positive cells. It can be speculated that this effect intensifies when more core sutures are used.

Myofibroblasts are not found in healthy tissues and make an appearance as a response to injury as part of the healing process. It has been shown that overactivity of myofibroblasts may lead to the formation of organ-specific fibrosis [21]. The presence of myofibroblasts per se is not to be regarded as directly prognostically unfavorable but is viewed as an important component of tissue reaction [21]. It is known that myofibroblasts resident in the gliding tissue around the tendon express *α*-SMA in contrast to fibroblasts resident within the tendon [21]. In this study, an increased *α*-SMA expression was detected at the three-week assessment subsiding to one-third after 8 weeks. In analogy to the findings in CAE staining, a tendency to higher levels of *α*-SMA expression was determined for the 4-strand core sutures. This difference in extracellular response between the 2- and 4-strand core suture repairs leveled out by the 8th week.

Doubling the core suture strands does not lead to more exposure of foreign material on the outside of the tendon as the suture material is completely enwrapped in the tendon. Other mechanisms must be held responsible for the build-up of the cellular and extracellular inflammatory response. The more difficult a suture technique is, the more the cut tendon ends are manipulated, increasing the surgical trauma. This can explain the increased cellular and extracellular inflammatory response.

The transferability of the results to humans is limited. The operated rabbits returned to unrestricted mobility immediately after coming out of anesthesia, so that the tendon repairs had to heal under maximum stress. Nevertheless, repair failure did not occur. A permanently disabling peritendinous scarring is probably prevented by the early active mobilization. However, an injured human being is more inclined to avoid tendon movements when the sliding of the tendon is impaired by inflammatory stimulus than a rabbit. The informative value of the study is limited by the omission of a biomechanical testing of the tendon repairs which was foregone in favor of a valid histological examination of mechanically not compromised tendons.

## 5. Conclusions

In this animal model it could be shown that increasing the number of suture strands leads to a temporarily limited enhancement of leukocyte infiltration and increased expression of *α*-SMA-positive cells, though not statistically significant. This potentially negative effect seems to be cancelled out by the benefits associated with a greater pull-out strength of the 4-strand suture. It could be proved that a 4-strand suture repair has a clear advantage over a 2-strand core suture in terms of gap formation in vivo.

## Figures and Tables

**Table 1:** Grading of gapping between cut tendon ends.

Grading	Distance between tendon ends
0	Adjacent tendon ends
1	Minimal distance, gap ≦ 1 mm
2	Gap ≧ 1 mm

**Table 2:** Tang et al. classification of peritendinous adhesion formation.

Stage	Characteristic features
1	No apparent adhesions, preserved peritendinous space
2	Minimal adhesions, peritendinous space preserved in most areas
3	Moderate adhesions, peritendinous space preserved in more than 50% of the tendon circumference
4	Severe adhesions, peritendinous space obliterated in more than 50% of the tendon circumference
5	Completely tethered tendon, no preserved peritendinous space

**Table 3:** Analysis of CAE-positive cell counts for 2- and 4-core suture repairs after 3 and 8 weeks.

Time of assessment (weeks after surgery)	Number of core suture throws	Count of CAE-positive cells (n/mm^2^) Mean value ± standard deviation
3	2	7.3 ± 2.2
3	4	8.9 ± 3.9
8	2	2.1 ± 0.7^*∗*^
8	4	1.9 ± 0.9^*∗*^

^*∗*^ *p* < 0.05 for difference between cell counts at assessment after 3 and 8 weeks.

**Table 4:** Analysis of *α*-SMA expression for 2- and 4-core suture repairs after 3 and 8 weeks.

Time of assessment (weeks after surgery)	Number of core suture throws	Count of formation of myofibroblasts (n/mm^2^) Mean value ± standard deviation
3	2	12.4 ± 3.8
3	4	17.3 ± 2.2
8	2	9.5 ± 2.4^*∗*^
8	4	10.3 ± 1.5^*∗*^

^*∗*^ *p* < 0.05 for difference between cell counts at assessment after 3 and 8 weeks.

## Abbreviations

*α*-SMA: Alpha smooth muscle actin
CAE: Chloroacetate esterase
cm: Centimeter
HE: Hematoxylin-eosin
kg: Kilogram
mm: Millimeter.
